# Entropy Driven Phase Transition in Polymer Gels: Mean Field Theory

**DOI:** 10.3390/e20070501

**Published:** 2018-06-30

**Authors:** Miron Kaufman

**Affiliations:** Physics Department, Cleveland State University, Cleveland, OH 44115, USA; m.kaufman@csuohio.edu

**Keywords:** crosslinking entropy, saturation, discontinuous phase transition

## Abstract

We present a mean field model of a gel consisting of *P* polymers, each of length *L* and *N_z_* polyfunctional monomers. Each polyfunctional monomer forms *z* covalent bonds with the 2*P* bifunctional monomers at the ends of the linear polymers. We find that the entropy dependence on the number of polyfunctional monomers exhibits an abrupt change at *N_z_* = 2*P*/*z* due to the saturation of possible crosslinks. This non-analytical dependence of entropy on the number of polyfunctionals generates a first-order phase transition between two gel phases: one poor and the other rich in poly-functional molecules.

## 1. Motivation

A polymer gel like polyacrylamide changes the volumes by a large factor of ~1000 when a small quantity of solvent like acetone is added to the solution or when the temperature is varied slightly. Central to the understanding of this phase transition are the covalent crosslinks [1,2,3,4,5] between the linear chains. Polymer chains, such as in hydroxypropylcellulose (HPC) immersed in a water solution, form hydrogen bonds with the water. As the temperature, the pH, or some other external condition is varied, there is a change in the strength of the hydrogen bonds that results in the formation or destruction of aggregates of crosslinked polymer chains. Light scattering experiments [6,7] are used to study the influence of the amount of crosslinkers on the properties of the gel phases. However, the theoretical work to date [1,2,3,4,5] explained the swelling transition as the sole result of the competition between the elastic energy of the gel network and the Flory-Huggins entropy and energy of linear polymers in solution, without accounting for the crosslinkers. Here, we study the mean-field theory of the thermodynamics of such a system that explicitly includes polyfunctional molecules that crosslink with bifunctional molecules at the ends of linear polymers.

The main goal of this work is to highlight the role of the crosslinking entropy and of the saturation effect that occurs when the number of polyfunctional molecules increases to become comparable to the number of polymers. With this in mind, and attempting not to clutter the issue, we present here numerical results for the special case of dry gels, no solvent, and we ignore the network elastic energy [1,2,3,4,5]. We find that the saturation of crosslinks induces a non-analytical dependence of entropy on the number of polyfunctional molecules, and consequently a strong first-order transition occurs even for zero energy. In other words, the crosslinking entropy drives a first-order transition.

In future work we will include all energy contributions (Flory-Huggins, elastic) and study the interplay of energy and entropy to achieve a more complete understanding of the thermodynamics of polymer gels. A mean field theory ignores the range of interactions, and this important aspect needs to be addressed by other techniques, such as Monte Carlo simulations or renormalization group calculations.

## 2. Model

The fundamental quantity of statistical mechanics that determines the thermodynamics is the partition function Ξ, which is the sum of the un-normalized Boltzmann probability function over all of the possible configurations. The logarithm of the partition function yields the thermodynamic potential *Ω*. Thus, the fundamental equation that gives the thermodynamic potential as a function of temperature, chemical potentials, etc. is determined.

We consider a lattice of *N* sites and volume *Nv* that is occupied by *N_z_* polyfunctionals, *N*_2_ bifunctionals supporting *P* linear chains each of length L, *N*_2_ = *P*(*L* + 1), and *N*_0_ solvent molecules. The total number of molecules is: *N* = *N_z_* + *N*_2_ + *N*_0_. The mean field [8,9] grand canonical partition function is:

(1) $$\Xi =\underset{N_{z},N_{0}}{\sum }\Gamma \left(N_{z},N_{0}\right){\left(\frac{1}{N}\right)}^{PL+B}\exp(-E\left(N_{z},N_{0}\right)/T)\exp\left(\frac{\mu_{z}N_{z}}{T}\right)\exp\left(\frac{\mu_{0}N_{0}}{T}\right)$$

where *Г* counts the number of configurations for a given *N_z_* and *N*_0_; *B* is the number of crosslinks formed between the *N_z_* polyfunctionals and the 2*P* bifunctionals at the ends of the chains; *μ_z_* is the chemical potential conjugated to the number of polyfunctionals, *μ*_0_ is the chemical potential conjugated to the number of solvent molecules, and we use units so that *k_B_* = 1. The mean field theory is exact for the equivalent neighbor lattice (also known as the Renyi-Erdos network). The equivalent-neighbor lattice has *N* vertices and *N*(*N* − 1)/2 edges connecting each pair of vertices. There is no distance dependence of the interactions and, as a result, the terms in the partition function of Equation (1) depend only on the number of molecules.

We assume that it is energetically favorable to establish as many crosslinks as possible. Hence: *B* = min(*zN_z_*, 2*P*). Each end of the *P* polymers can accept one crosslink, and the *N_z_* polyfunctionals can each accept *z* bonds. The energy is expressed as a quadratic function of the three numbers of molecules: *N*_0_, *N_z_*, and *N*_2_:

(2) $$\;\frac{E}{T}=\chi_{0}N_{0}(1-N_{0}/N)+\chi_{z}N_{z}\left(1-\frac{N_{Z}\;}{N}\right)+\chi_{2}N_{2}\left(1-N_{2}/N\right)+\;\chi_{0z}N_{0}N_{z\;}/N+\chi_{2z}N_{2}N_{z\;}/N+\chi_{02}N_{2}N_{0\;}/N$$

An important contribution to the energy that needs to be included in a complete model of gels is the Flory-Rehner elastic energy [10] of the gel network. One can also include the concentration dependence of the various χ coefficients (see Hirotsu in [1,2,3,4,5]). In Equation (2) above, we only listed terms that are quadratic in densities.

The combinatorical factor *Γ* = exp(*S*) and *S* is the entropy. There are three contributions to the entropy *S*: (1) the entropy of mixing the *N_z_* polyfunctionals with the *N*_2_ byfunctionals and with the *N*_0_ solvent molecules; (2) the Flory-Huggins entropy [8,9,10] associated with arranging P linear chains on the byfunctionals, *N*_2_ = *P*(*L* + 1); (3) the crosslinking entropy associated with establishing covariant bonds between the 2*P* ends of the chains and the *N_z_* polyfunctionals. The latter entropy exhibits non-analytical behavior as a function of *N_z_* at 2*P*/*z*, which triggers a phase transition.

In the thermodynamic limit (large *N*), the partition function $=\sum e^{N\Psi }$ is approximated by the largest term [8,9] in the sum. Then, the grand-canonical thermodynamic potential *Ω* is:

(3) $$-\frac{\Omega }{T}=\ln\left(\right)=\max\left[S-\left(PL+B\right)\ln\left(N\right)+\frac{-E\left(N_{z},N_{0}\right)+\mu_{z}N_{z}+\mu_{0}N_{0}}{T}\right]$$

Using the Euler equation of thermodynamics, *E* = *TS* − *pV* + *μ_z_N_z_* + *μ*_0_*N*_0_, one can relate the thermodynamic potential, *Ω* = *E* − *TS* − *μ_z_N_z_* − *μ*_0_*N*_0_, to the osmotic pressure:

Ω= −pNv

where *v* is the volume per molecule. The Euler equation is the direct consequence of the extensiveness of energy and entropy. Equation (3) implies:

(4) $$\frac{pv}{T}=\;\max\left[\Psi \right]$$

where

(5) $$\Psi =\hat{s}+\frac{-e\left(n_{z},n_{0}\right)+\mu_{z}n_{z}+\mu_{0}n_{0}}{T},s=S/N,p=P/N,b=B/N,n_{z}=N_{z}/N,n_{0}=N_{0}/N,\mathrm{and}\hat{s}=s-\left(pL+b\right)\ln\left(N\right)$$

## 3. Entropy

The entropy is the sum of three physically distinct contributions: *S* = *S*_1_ + *S*_2_ + *S*_3_.

The entropy of mixing the *N_z_* polyfunctionals, the *N*_2_ bifunctionals, and the solvent molecules *N*_0_:

(6) $$e^{S_{1}}=\;\frac{N!}{N_{0}!N_{2}!N_{z}!}$$

Here, *N* = *N*_0_ + *N*_2_ + *N_z_*.

The entropy that is associated with arranging *P* linear chains, each of length *L*, on the set of *N*_2_ = *P*(*L* + 1) bifunctionals is [8,10]:

(7) $$e^{S_{2}}=\;\frac{N_{2}!}{P!2^{P}}$$

Previous models [1,2,3,4,5] have accounted only for the above two contributions to the entropy: Flory-Huggins entropy. The main contribution of this paper is the calculation of the entropy *S*_3_ that is associated with establishing covariant bonds between the 2*P* byfunctionals at the ends of the chains and the *N_z_* polyfunctionals. One gets different formulas depending on relative sizes of 2*P* and *zN_z_*.

We assume that energetically it is favorable to generate as many covalent bonds as possible between the *N_z_* crosslinkers (each accepts *z* bonds) and the 2*P* bifunctionals at the ends of the linear polymers (each accepts one bond). The number of configurations is obtained by using combinations C(*N*, *K*) = *N*!/*K*!(*N* − *K*)! of *N* objects that are taken in groups of *K* objects.

If 2*P* > *zN_z_*, the number of configurations of crosslinking bonds is:

(8) $$e^{S_{3}}=\;C\left(2P,z\right)*C\left(2P-z,z\right)*\ldots *C\left(2P-\left(Nz-1\right)z,z\right)\;=\frac{\left(2P\right)!}{\left(2P-zN_{z}\right)!{\left(z!\right)}^{N_{z}}}$$

Here, *C*(2*P*, *z*) counts the number of configurations of z bonds coming out of one polyfunctional and each bond connecting to an end of a linear polymer. The second term counts the same type of bond configurations from a second polyfunctional and bonds ending on *z* of the 2*P* − *z* free ends of polymers. This process continues until all polyfunctionals are saturated: there are *N_z_* terms in the product.

If 2*P* < *zN_z_*, the number of configurations of crosslinking bonds is

(9) $$e^{S_{3}}=C\left(2P,z\right)*C\left(2P-z,z\right)*\ldots *C\left(2P-\left(\frac{2P}{z}-1\right)z,z\right)*C\left(N_{z},\frac{2P}{z}\right)=\;\;\;=\frac{\left(2P\right)!(N_{z})!}{{\left(z!\right)}^{\frac{2P}{z}}\left(\frac{2P}{z}\right)!\left(N_{z\;}-\;\frac{2P}{z}\right)!}$$

Here, the process of counting is similar. There are 2*P*/*z* factors counting the crosslinks configurations: from each polyfunctional, *z* bonds connect to 2*P* ends of linear polymers. The last factor in Equation (9) counts the number of groups of 2*P*/*z* polyfunctionals that can be formed out of the available *N_z_* molecules.

The entropy per molecule *s* = *S*/*N* is obtained by using the equations above and the Stirling approximation. We find:

(10) $$\hat{s}=-n_{0}\ln\left(n_{0}\right)-n_{z}\ln\left(n_{z}\right)-\left(2p-zn_{z}\right)\ln\left(2p-zn_{z}\right)+p\ln\left(p\right)-\left(L-\ln\left(2\right)\right)p-\left(z+\ln\left(z!\right)\right)n_{z}\;\;\;\;\;\;\;\;\;\;\;\;\;\;\;\;\;\;\;\;\;\;\;\;\;\;\;\;\;\;\;\;\;\;\;\;\;\;\;\;\;\;\;\;\;\;\mathrm{if}\;\;zn_{z}<2p$$

(11) $$\hat{s}=-n_{0}\ln\left(n_{0}\right)-\left(n_{z}-\frac{2p}{z}\right)\ln\left(n_{z}-\frac{2p}{z}\right)+\frac{z-2}{z}p\ln\left(p\right)-\left(L-\ln\left(2\right)+2+\frac{2}{z}\ln\left(2\left(z-1\right)!\right)\right)p\;\;\;\;\;\;\;\;\;\;\;\;\;\;\;\;\;\;\;\;\;\;\;\;\mathrm{if}\;\;zn_{z}>2p$$

## 4. Results

We solved numerically the maximization Equation (4) for the special case of no solvent molecules $n_{0}=0$ and for $\chi_{2}=\chi_{2z}=0$. These parameter values are chosen to highlight the role of the crosslinking entropy in triggering the first-order transition, even for zero energy. This is a novel entropic effect that is not included in previous studies [1,2,3,4,5]. This model exhibits strong first-order transitions originating in the non-analytical behavior of the entropy as a function of densities at 2*P* = *zN_z_*. Its physical origin is the transition from satisfying all the byfunctionals if 2*P* < *zN_z_* to satisfying all of the polyfunctionals if *zN_z_* < 2*P*. The crossover condition *zN_z_* = 2*P* corresponds to a density of polyfunctionals of ${n_{z}}^{*}=\frac{1}{1+\frac{z}{2}\left(L+1\right)}$. Hence for large *z*, *z* >> 1, the crossover occurs for very low volume densities of polyfunctionals, while for smaller *z*~1 (such as for water), the crossover occurs at larger volume densities of crosslinkers. The numerical results below are for *z* = 3 and *L*= 3.

**Figure 1:**
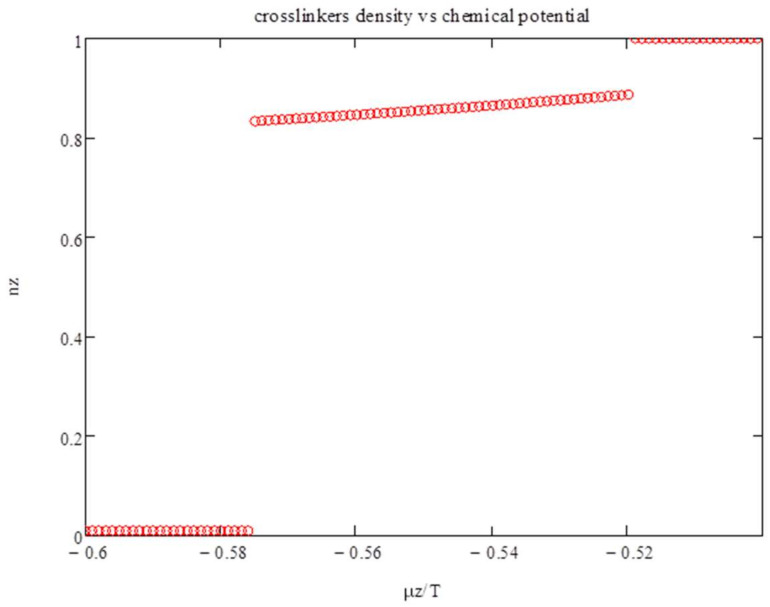
The density of polyfunctionals *n_z_* versus the chemical potential *μ_z_*/*T* for zero energy.

To demonstrate this, we first consider the model with zero energy: $\chi_{z}=0$, i.e., at very high temperatures. In Figure 1, we show the density of polyfunctionals n_z_ as a function of the chemical potential *μ_z_*/*T*. Phase transitions occur around *μ_z_*/*T*~−0.5. Two first-order transitions are exhibited: from *n_z_*~0 to *n_z_*~0.9 to *n_z_*~1.

**Figure 2:**
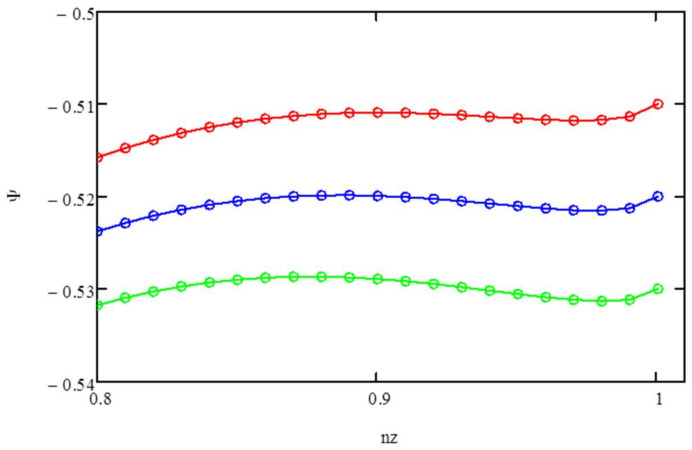
*Ψ* vs. *n_z_*. There is a phase transition between *nz*~0.89 and *nz* = 1 for *μ_z_*/*T* = −0.52 (middle curve). For *μ_z_*/*T* = −0.53 (lower curve), the maximum of *Ψ* is at *n_z_* = 0.89. For *μ_z_*/*T* = −0.51 (upper curve), the maximum in ***Ψ*** occurs at *n_z_* = 1.

The latter transition occurs at *μ_z_*/*T* ~ −0.52. To understand it, we show in Figure 2, ***Ψ*** vs. *n_z_* for *μ_z_*/*T* = −0.53 (lower curve), −0.52 (middle curve), and −0.51 (upper curve). The maximum in ***Ψ*** occurs at *n_z_* ~ 0.89 and at 1 for *μ_z_*/*T* ~ −0.52. 

**Figure 3:**
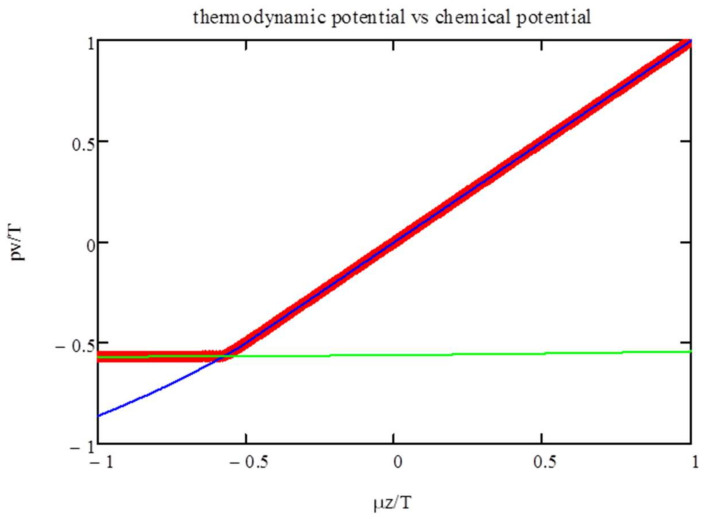
*pv*/*T* vs. *μ_z_*/*T* for zero energy; the entropic crossover between *zn_z_* < 2*p* and *zn_z_* > 2*p* is responsible for the transition. The two lines correspond to the entropy formula, Equations (10) and (11). In the thermodynamic limit, the maximization procedure, Equation (4), choses for a given chemical potential the higher osmotic pressure, heavy curve.

In Figure 3, we show the thermodynamic potential, i.e., max(***Ψ***), as a function of the chemical potential. The graph also includes the branches corresponding to the two formulas for the entropy (Equations (10) and (11)). This shows that the phase transitions are induced by the changeover in the entropy dependence on the polyfunctionals’ density.

**Figure 4:**
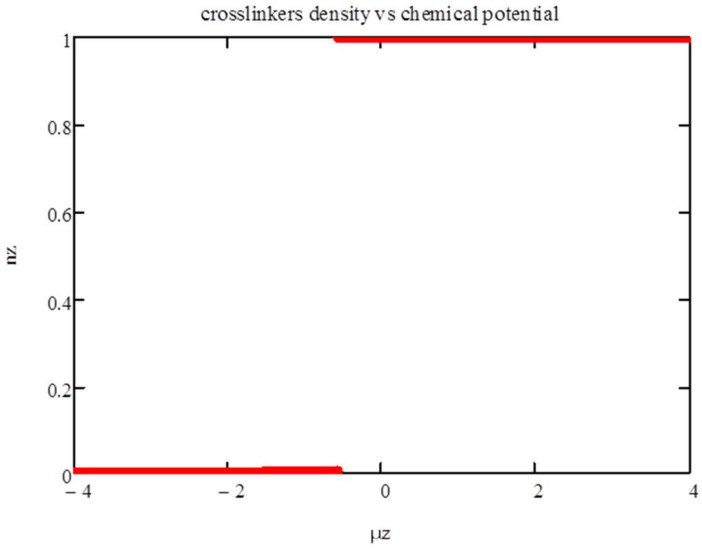
Density of polyfunctionals *n_z_* versus chemical potential *μ_z_* for *χ_z_* = 1 and *T* = 1.

**Figure 5:**
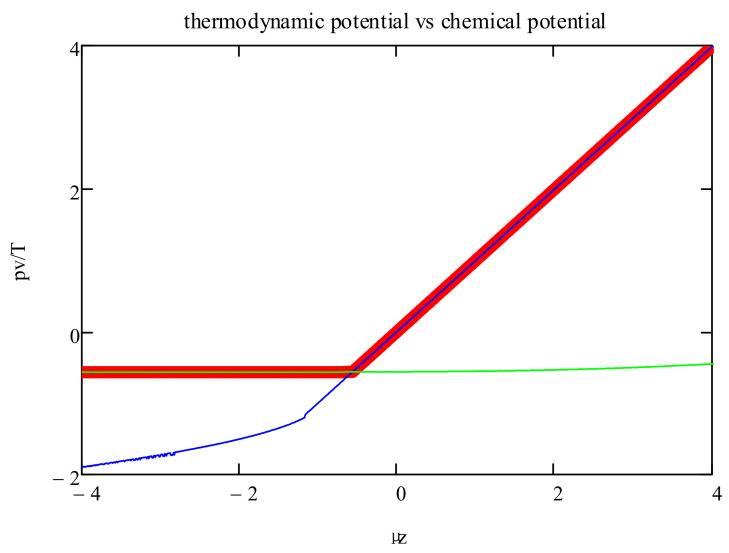
*pv*/*T* vs. *μ_z_* for *χ_z_* = 1 and *T* = 1. The two lines correspond to the entropy formula, Equations (10) and (11). In the thermodynamic limit, the maximization procedure, Equation (4), choses for a given chemical potential the higher osmotic pressure, heavy curve.

Next, we consider $\chi_{z}=1$. We show in Figure 4 and Figure 5 the isotherms for *T* = 1. There is a phase transition from a phase with very few polyfunctionals to a phase with *n_z_* ~ 1 at *μ_z_* ~ −0.575.

Figure 5 shows the thermodynamic potential vs. the chemical potential. It confirms that the first-order transition is triggered by the entropic crossover from *n_z_* < 2*P*/*z* to *n_z_* > 2*P*/*z*. The presence of the energy term *χ_z_n_z_*(1 − *n_z_*) strengthen this transition, as this energy is minimized at *n_z_* = 0 and 1. As a result, the intermediate phase that is apparent at zero energy (see Figure 1) does not show up here.

## 5. Conclusions

The importance of crosslinks that are formed between polymers and other chemicals in solutions was recognized [1,2,3,4,5] as central to understanding phase transitions (such as swelling) in polymeric systems. In this work, we have explicitly calculated the crosslinking entropy. We have shown that the entropy that is associated with the formation of crosslinking bonds between the ends of linear polymers and the polyfunctional molecules crosses over from one dependence for 2*P* < *zN_z_* (when 2*P* bonds are formed) to another one for *2P* > *zN_z_* (when *zN_z_* bonds are formed). The crossover results in strong first-order transitions between a phase poor in crosslinkers and a phase with a high concentration of crosslinkers. The important result demonstrated in this work is that the crosslinking entropy that measures the number of possibilities of establishing bonds between the ends of the linear polymers and the polyfunctional molecules, drives a strong first order transition.

This model can be expanded to include the elasticity energy of the linear polymers, which was considered by Tanaka and Hirotsu [1,2,3,4,5]. We will also analyze the phase diagram in the five-dimensional space of interactions, three couplings, and two chemical potentials, which is quite similar to the three-component model [11]. Furthermore, there is experimental evidence that the polymers are polydisperse as opposed to monodisperse (as was assumed here). The influence of the size distribution of polymers on the phase transitions is expected [8,10] to be quite interesting and will be considered. The role of the density of crosslinks on the thermodynamic properties can be analyzed by varying the functionality parameter *z*. This is relevant to slightly crosslinked gels, *z* >> 1, that are known to be superabsorbent [12].

The mean-field theory is exact for the equivalent-neighbor lattice [8,9] where the interactions are infinitely ranged: they are independent of distance. The success of the Flory-Huggins [10] theory of polymer solutions may be due to its realizability (in Berker’s sense [13,14]), i.e., it is exact for the equivalent neighbor lattice model, though it is approximate for the three dimensional system. This ensures that the thermodynamics laws are satisfied [13,14]. To introduce a distance in the model, one considers energy terms such as *J_ij_n_i_n_j_*, where *n_i_* is one if a particle is present at location *i* or zero if it is absent, and the coupling *J* depends on the distance between *i* and *j*. The mean-field theory is generally reliable in predicting qualitatively correct phase diagrams. However, for realistic finite-range interactions, the critical exponents [15] are generally modified, and the type of transition [16]—first- or second-order—may differ. The role of the range of interactions can be studied by means of Monte-Carlo simulations [17,18] and renormalization group calculations [19].
